# Reducing Effect of Saikosaponin A, an Active Ingredient of *Bupleurum falcatum*, on Intake of Highly Palatable Food in a Rat Model of Overeating

**DOI:** 10.3389/fpsyt.2018.00369

**Published:** 2018-08-13

**Authors:** Paola Maccioni, Federica Fara, Gian Luigi Gessa, Mauro A. M. Carai, Young-Won Chin, Jung Hwan Lee, Hak Cheol Kwon, Giancarlo Colombo

**Affiliations:** ^1^Neuroscience Institute, Section of Cagliari, National Research Council of Italy, Monserrato, Italy; ^2^Cagliari Pharmacological Research s.r.l., Cagliari, Italy; ^3^College of Pharmacy, Dongguk University-Seoul, Goyang, South Korea; ^4^Korea Institute of Science and Technology, Gangneung Institute of Natural Products, Gangneung-si, South Korea

**Keywords:** saikosaponin A, *Bupleurum falcatum L*., rimonabant, overeating, palatable food, rats

## Abstract

Recent lines of experimental evidence have indicated that saikosaponin A (SSA)—a bioactive ingredient of the medicinal plant, *Bupleurum falcatum L*.—potently and effectively reduced operant self-administration of chocolate and reinstatement of chocolate-seeking behavior in rats. The present study was designed to assess whether the protective properties of SSA on addictive-like, food-related behaviors generalize to a rat model of overeating of palatable food. To this end, rats were habituated to feed on a standard rat chow for 3 h/day; every 4 days, the 3-h chow-feeding session was followed by a 1-h availability of highly palatable, calorie-rich Danish butter cookies or Oreo chocolate cookies. Even though fed, rats consumed large amounts of cookies; intake of calories from cookies (consumed in 1 h) was even larger than that of calories from chow (consumed in 3 h). SSA (0, 0.25, 0.5, and 1 mg/kg, i.p.) was administered 10 min before cookie presentation. Treatment with SSA resulted in a dose-related decrease in intake of both butter and chocolate cookies. Administration of the cannabinoid CB_1_ receptor antagonist/inverse agonist, rimonabant (0, 0.3, 1, and 3 mg/kg, i.p.; tested as reference compound), produced a similar reduction in intake of butter cookies. These results (a) contribute to the set-up and validation of a rat model of overeating, characterized by the intake of large amounts of unnecessary calories and (b) provide an additional piece of evidence to the anorectic profile of SSA in rats.

## Introduction

Saikosaponin A (SSA) is one of the major ingredients of the plant *Bupleurum falcatum L*., the roots of which are largely used in traditional Chinese, Korean, and Japanese medicine for the treatment of various diseases, including psychiatric and neurological disorders [see (1)]. Recent lines of experimental evidence have demonstrated that treatment with SSA potently and selectively suppressed intravenous self-administration of morphine (2) and cocaine (3) and oral self-administration of alcohol (4) in rats; together, these data suggest that SSA may interfere with the brain mechanisms underlying the reinforcing and motivational properties of drugs of abuse.

Substances of abuse and natural stimuli, including palatable food, share common neural substrates mediating their rewarding, reinforcing, and stimulating properties [see (5)]. This remarkable overlap includes the brain “reward” dopamine pathway, the neurons of which are activated by both drugs of abuse (*via* direct pharmacological actions) and palatable foods (*via* fast sensory inputs) [see (6)]. Testing SSA on self-administration of a palatable food was therefore consequential: as predictable, treatment with SSA, tested at doses identical to those found to effectively suppress morphine, cocaine, and alcohol self-administration (2–4), suppressed self-administration of and reinstatement of seeking behavior for a highly palatable chocolate-flavored beverage in rats (7). Notably, the chocolate-flavored beverage used in the above study possesses reinforcing and motivational properties strong enough to generate addictive-like behaviors in rats [see (8)].

With the intent of characterizing further the ability of SSA to affect behaviors motivated by palatable food, the present study investigated the effect of treatment with SSA on overeating of highly palatable, calorie-rich cookies in pre-fed rats. To this end, rats were habituated to a daily regimen of 21 h of food deprivation followed by 3 h of free access to regular chow. The deprivation period led rats to consume large amounts of chow over the 3-h period; this chow intake was large enough to feed the rats, as indicated by the progressive increase in rat body weight. Every 4 days, the daily chow-feeding session was followed by a 1-h exposure to Danish butter cookies or Oreo chocolate cookies. Under this regimen, rats displayed cookie hyperphagia and large intake of unnecessary calories: calories from cookies (consumed in 1 h) were even higher than those from chow (consumed in 3 h). Danish butter cookies and Oreo chocolate cookies were chosen because often employed in rodent studies investigating the neurobiological and pharmacological bases of food addiction [e.g., (9, 10)]. SSA was administered at the end of the 3-h exposure to chow and immediately before the start of the 1-h period of access to cookies.

The results of preliminary experiments conducted using the above procedure indicated that chow intake was steadily and markedly reduced in the first daily feeding session following cookie exposure (this laboratory, unpublished results); notably, these reductions in chow intake occurred in spite of the 20 h of food deprivation elapsing between cookie removal and chow presentation. We hypothesized that this day-after hypophagia could be the result of the caloric overload attained the day before with cookie overeating; alternatively, it could be due to a self-restriction of chow feeding enacted by rats in anticipation of access to cookies, similar to the anticipatory self-hypophagia of regular chow observed in mildly food-deprived rats exposed to two brief, contiguous periods of access to chow first and chocolate pellets later (11). To experimentally address this research question, we compared the chow intake of 2 groups of rats that, the day before, (a) had access to butter cookies in a 1-h feeding session or (b) were forcedly administered, *via* intragastric gavage, an amount of butter cookies (finely granulated and then softened in water) equivalent to that consumed freely.

Finally, the cannabinoid CB_1_ receptor antagonist/inverse agonist, rimonabant, was included in the experimental design as reference compound with the 2-fold intent of providing (a) pharmacological validation of this experimental model of overeating and (b) a touchstone for the effect of treatment with SSA. Rimonabant was chosen as reference compound as it has repeatedly been reported to suppress multiple behaviors related to highly palatable food in rats [e.g., (12–16)].

## Materials and methods

This study was carried out in accordance with the recommendations of the European (Directive no. 2010/63/EU of September 22, 2010) and Italian (Legislative Decree no. 26 of March 4, 2014) laws on the “Protection of animals used for scientific purposes.” The protocol was approved by the Ethics Committee (*Organismo preposto al benessere animale*) of the University of Cagliari, Italy.

### Animals

The present study employed adult, male Wistar rats (Harlan Laboratories, San Pietro al Natisone, Italy). Starting from the age of 70 days, rats were housed individually in standard cages with wood chip bedding. The animal facility was under an inverted 12:12 h light-dark cycle (lights on at 9:00 p.m.), constant temperature of 22 ± 2°C, and relative humidity of ~60%. Rats were extensively habituated to handling, intragastrical infusion (Experiment 1), and intraperitoneal injection (Experiments 2 and 3). Each experiment used an independent set of rats.

### Plant material and extraction procedure of SSA

The root of *Bupleurum falcatum* was purchased from Korean herbal market in Yeongcheon, Republic of Korea. The dried root was sliced and extracted with 70% ethanol (4 × 7.0 L) using a refluxing extraction method at 50°C. The extract was suspended in water (1.0 L) and then partitioned with dichloromethane (3 × 1.0 L) and 1-butanol (3 × 1.0 L) sequentially. The 1-butanol soluble fraction (46.5 g) was fractionated on Diaion HP-20 column (methanol/water, 1:4, 2:3, 3:2, 4:1, and 1:0; 4 L for each eluent) to give 5 fractions (F1 to F5). F5 (4.8 g) was separated by preparative HPLC with a YMC Pack ODS-A column (acetonitrile/water, 13:37 to 2:3, flow rate: 10.0 mL/min) and finally purified by preparative HPLC with RI detector using a Phenomenex Luna C18(2) column (methanol/water, 67:33, flow rate: 4.0 mL/min) to afford SSA (200 mg, >95%).

### Experimental procedure

**Figure 1:**
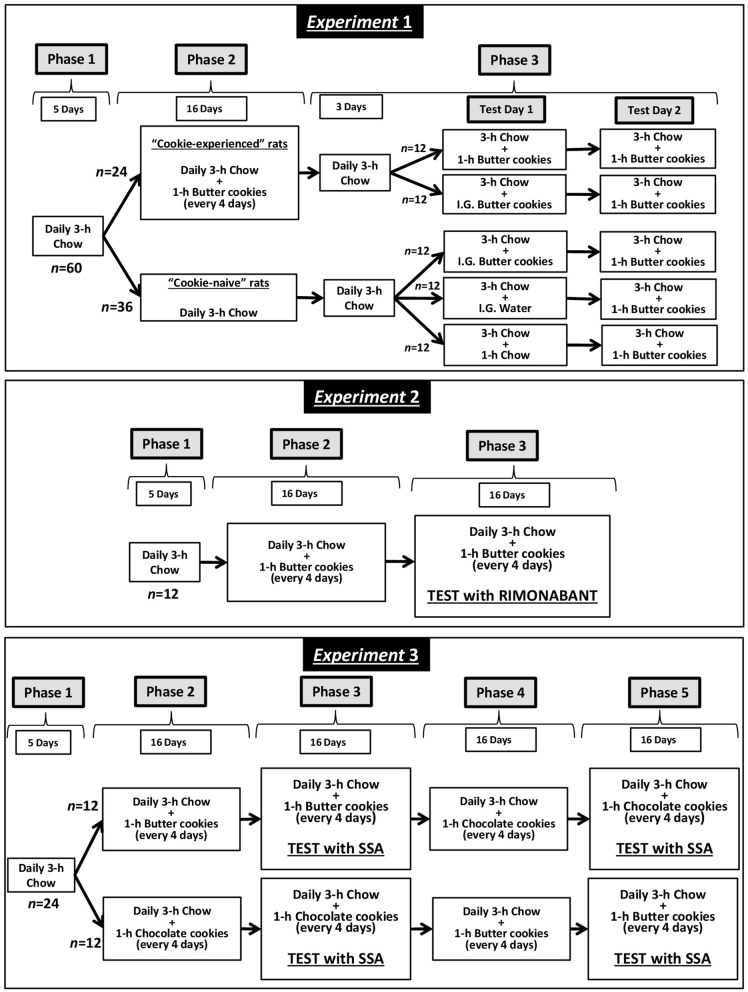
Schematic representation of the experimental design.

Schematic representation of the design of Experiments 1 (set-up of the experimental procedure), 2 (test with rimonabant), and 3 (test with SSA) is given in Figure 1.

#### Experiment 1: set-up of the experimental procedure

Starting from the age of 70 days, rats (*n* = 60) were habituated to feed a standard rat chow (Harlan Laboratories, San Pietro al Natisone, Italy) for 3 h/day (coinciding with the first 3 h of the dark phase); no food was available during the remaining 21 h. Tap water was available 24 h/day. This procedure was maintained for 5 consecutive days (Phase 1), leading to stable daily intakes of chow over the 3-h feeding period. The duration of the daily chow-feeding session was selected on the basis of results of preliminary experiments indicating that it was long enough to allow adult, male rats to feed amounts of food fully sufficient to maintain stable, or even increase, body weights (this laboratory, unpublished results).

Subsequently, rats were divided into 2 groups. Both rat groups were kept under the above feeding regimen (daily 3-h chow-feeding sessions followed by 21-h food deprivation). Rats of the first group (*n* = 24) were also exposed to 1-h sessions with access to butter cookies (Kelsen Group, Snede, Denmark; “cookie-experienced” rats); these sessions occurred (i) every 4 days and (ii) immediately after the end of the 3-h chow-feeding session. This phase (Phase 2) lasted 16 consecutive days and included 4 feeding sessions with butter cookies (during which cookie intake stabilized). Conversely, rats of the second group (*n* = 36) were not exposed to butter cookies (“cookie-naive” rats).

Phase 3 comprised 3 additional daily 3-h chow-feeding sessions *plus* 2 consecutive test days. On the first test day (Test Day 1), “cookie-experienced” rats were divided into 2 subgroups of *n* = 12. At the end of the 3-h chow-feeding session, rats of one subgroup were exposed to a regular 1-h cookie-feeding session, while the rats of the other subgroup were administered intragastrically with 9 g/rat butter cookies, finely granulated and then softened in water. To minimize rat discomfort possibly associated with intragastric infusion of a large volume and to better reproduce the pattern of cookie intake during the 1-h cookie-feeding session, the amount of cookies was divided into 2 different administrations (volume of each infusion: 9 ml/rat) occurring 30 min apart (immediately after the end of the 3-h chow-feeding session and 30 min later). The amount of butter cookies to be administered was calculated as to be identical to the mean amount of cookies consumed by the cookie-experienced rats in the 2 sessions preceding the test.

On Test Day 1, “cookie-naive” rats were divided into 3 subgroups of *n* = 12. At the end of the 3-h chow-feeding session, rats of the first subgroup were administered intragastrically with 9 g/rat butter cookies (cookie administration was performed as described above). Rats of the second subgroup were administered intragastrically with 9 ml/rat tap water twice (30 min apart). Rats of the third subgroup were exposed to an additional 1-h chow-feeding session.

On the second test day (Test Day 2), all rats (“cookie-experienced” and “cookie-naive”) were exposed to a 3-h chow-feeding session followed by a 1-h cookie-feeding session.

#### Experiment 2: investigation of the effect of rimonabant

The initial phases of Experiment 2 were identical to those of Experiment 1. Briefly, 70-day-old rats (*n* = 12) were habituated to chow-feeding for 3 h/day and 5 consecutive days (Phase 1). Rats were then kept under the 3-h feeding regimen for additional 16 consecutive days, and exposed to 4 1-h sessions with butter cookies (Phase 2); these cookie-feeding sessions occurred (i) every 4 days and (ii) immediately after the end of the 3-h chow-feeding session.

After 3 additional daily 3-h chow-feeding sessions, rats underwent testing with rimonabant (Phase 3). Test sessions with rimonabant were conducted every 4 days and all doses of rimonabant were tested in each rat under a Latin-square design. Four consecutive daily 3-h chow-feeding sessions elapsed between test sessions with rimonabant. Rimonabant (Sanofi-Aventis, Montpellier, France) was suspended in saline with 1% (v/v) Tween 80 and administered intraperitoneally (injection volume: 2 ml/kg), at the doses of 0, 0.3, 1, and 3 mg/kg, at the time of chow removal and 20 min before cookie presentation. Rimonabant dose-range was selected on the basis of results of previous studies demonstrating its ability to reduce several behaviors related to palatable food, including consumption (13) and operant self-administration (15) of a chocolate-flavored beverage, in Wistar rats.

#### Experiment 3: investigation of the effect of SSA

The initial phases of Experiment 3 were identical to those of Experiment 1. Briefly, 70-day-old rats (*n* = 24) were habituated to chow-feeding for 3 h/day and 5 consecutive days (Phase 1). Rats were then divided into 2 groups of *n* = 12, kept under the 3-h feeding regimen for additional 16 consecutive days, and exposed to 4 1-h sessions with butter cookies or Oreo chocolate cookies (Mondelez, Milan, Italy; Phase 2); these cookie-feeding sessions occurred (i) every 4 days and (ii) immediately after the end of the 3-h chow-feeding session.

After 3 additional daily 3-h chow-feeding sessions, rats of both groups (butter cookies and chocolate cookies) underwent testing with SSA (Phase 3). Test sessions with SSA were conducted every 4 days and all doses of SSA were tested in each rat under a Latin-square design. Four consecutive daily 3-h chow-feeding sessions elapsed between test sessions with SSA. SSA was suspended in saline with 1% (v/v) Tween 80 and administered intraperitoneally (injection volume: 2 ml/kg), at the doses of 0, 0.25, 0.5, and 1 mg/kg, at the time of chow removal and 10 min before cookie presentation. SSA dose-range was selected on the basis of results of a previous study (7) demonstrating its ability to reduce operant self-administration of a chocolate-flavored beverage in Wistar rats. Time of SSA treatment was chosen according to data on SSA pharmacokinetics indicating that it has a half-life of ~30 min after intravenous injection in rats (17, 18). This short half-life ruled out any possible carry-over effect of SSA on feeding behavior on the day after SSA injection.

Rats were then exposed to an additional series of 16 consecutive daily 3-h chow-feeding sessions and 4 1-h cookie-feeding sessions (Phase 4); cookie type was inverted between the 2 rat groups, so that rats previously exposed to butter cookies were now exposed to chocolate cookies, and rats previously exposed to chocolate cookies were now exposed to butter cookies. Rats of both groups underwent another test with SSA, again under a Latin-square design, with daily 3-h chow-feeding sessions elapsing between test sessions with SSA (Phase 5). SSA was injected as described above. Since there was no difference in chow intake between Phases 4 and 5, at the end of Phase 5 data from SSA testing were cumulated to a final sample size of *n* = 24.

## Measured variables and data analysis

In each daily 3-h chow-feeding session, measured variables were as follows: (a) chow intake (expressed in g/kg); (b) calorie intake from chow (expressed in kcal/kg and calculated from the 3.30 kcal/g-value of the standard chow). Chow intake was recorded by weighing (with a 0.01-g accuracy) food pellets immediately before and immediately after the session.

In each 1-h feeding (cookies or chow) session, measured variables were as follows: (a) cookie or chow intake (expressed in g/kg); (b) calorie intake from cookies (expressed in kcal/kg and calculated from the 5.19 and 4.76 kcal/g-value of butter and chocolate cookies, respectively) or chow (expressed and calculated as described above); (c) water intake (expressed in ml/kg). Cookie, chow, and water intake was recorded by weighing (with a 0.01-g accuracy) cookies, food pellets, and water bottles immediately before and immediately after the session.

In Experiment 1, data on each variable were analyzed by 1- or 2-way ANOVA for repeated measures, followed by the Tukey or Šidák test for *post hoc* comparisons.

Data on the effect of treatment with rimonabant (Experiment 2) and SSA (Experiment 3) on each variable were analyzed by 1-way ANOVA for repeated measures, followed by Tukey test for *post hoc* comparisons.

## Results

**Figure 2:**
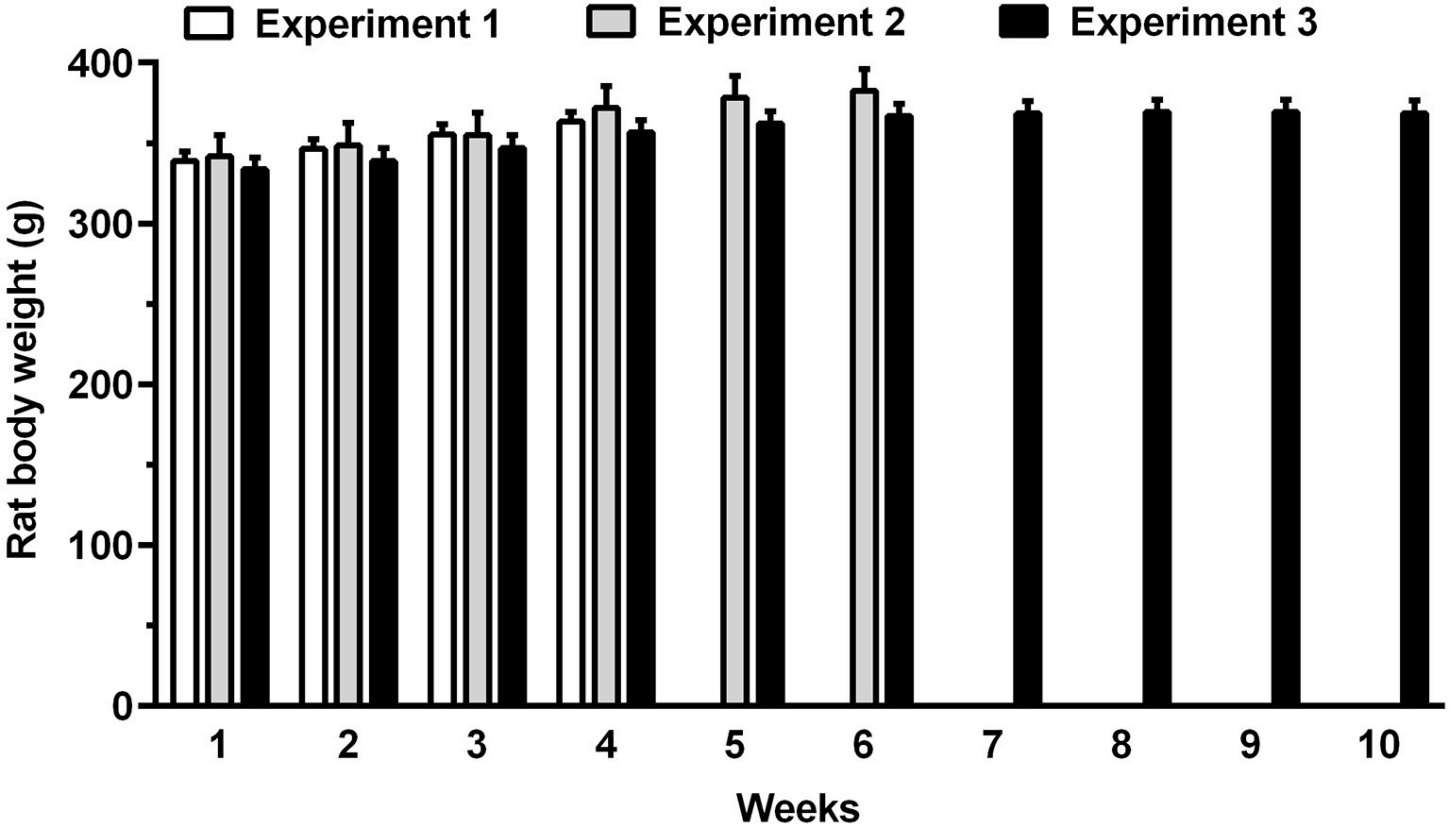
Body weight of rats allocated to Experiments 1–3 over the entire course of the study. Rat body weight was recorded weekly. Each bar is the mean ± SEM of *n* = 60 (Experiment 1), *n* = 12 (Experiment 2), and *n* = 24 (Experiment 3) rats.

The daily 3-h chow-feeding regimen was fully adequate to meet the daily caloric needs of rats, as mean body weight of all rat groups tended to increase over the course of the study (Figure 2). In Experiment 1, body weight gain was identical in “cookie-experienced” and “cookie-naive” rat groups.

### Experiment 1: set-up of the experimental procedure

**Figure 3:**
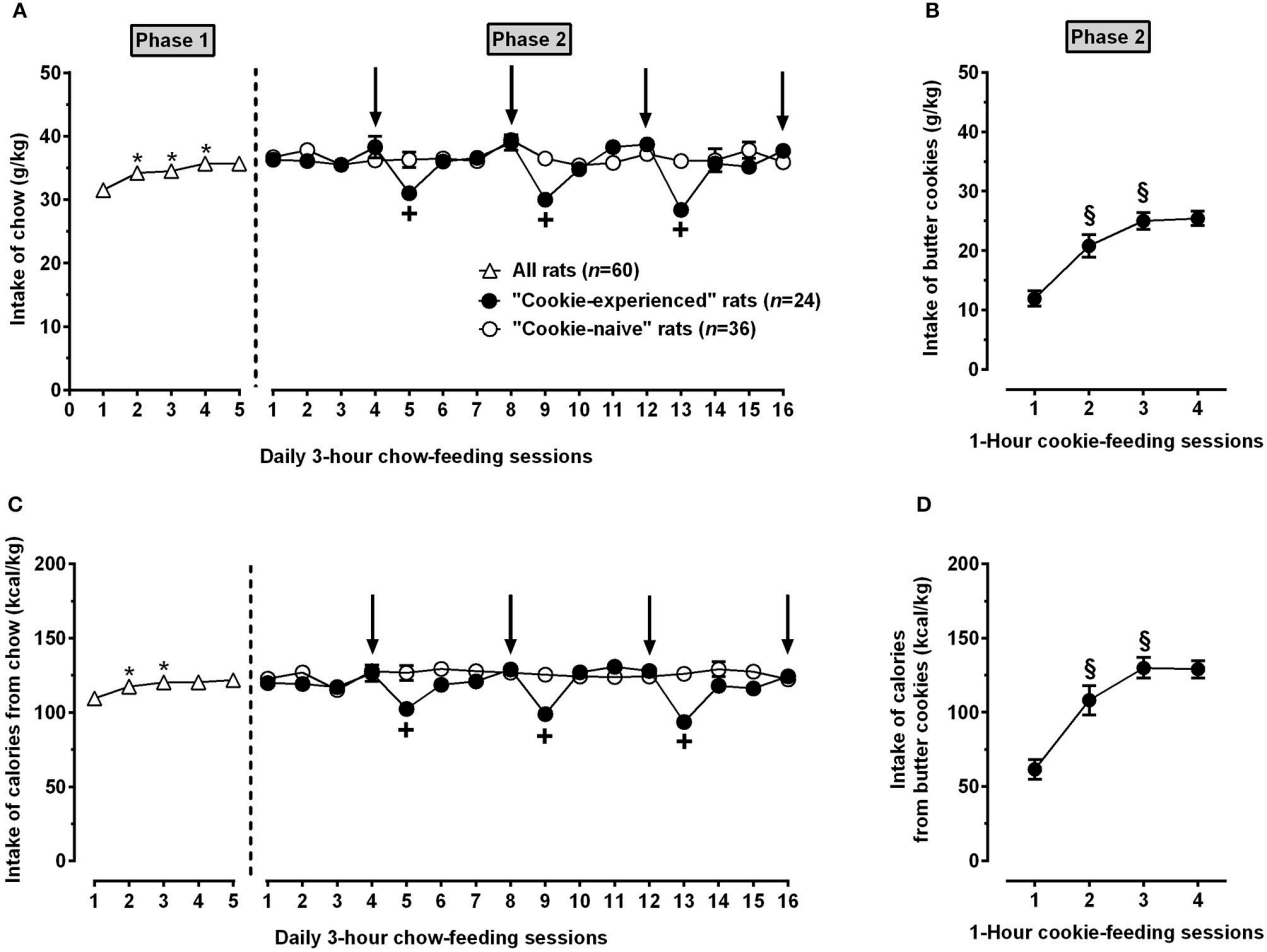
Intake of chow **(A)** and butter cookies **(B)** as well as intake of calories from chow **(C)** and butter cookies **(D)** in rats exposed to Phases 1 and 2 of Experiment 1. In Phase 1 (lasting 5 consecutive days), all rats (*n* = 60) were exposed to a regimen of daily 3-h chow-feeding sessions followed by 21 h of food deprivation. In Phase 2 (lasting 16 consecutive days), all rats were kept under the above feeding regimen; rats were divided into 2 groups and exposed (“cookie-experienced” rats; *n* = 24) or not (“cookie-naive” rats; *n* = 36) to 4 1-h cookie-feeding sessions occurring immediately after the end of the chow-feeding session (arrows indicate the days in which the cookie-feeding sessions took place). Each point is the mean ± SEM of *n* = 24-60 rats. *P* < 0.05 in comparison to the day before (Tukey test); ^+^*P* < 0.05 in comparison to the “cookie-naive” rat group in the same chow-feeding session (Šidák test); ^§^ *P* < 0.05 in comparison to the day before (Tukey test).

In Phase 1, rats became rapidly habituated to the daily 3-h chow-feeding regimen, reaching stable intakes of chow (Figure 3A) and calories (Figure 3C) from the 3rd or 4th chow-feeding session [chow intake: *F*_(4, 236)_ = 6.62, *P* < 0.0005; calorie intake: *F*_(4, 236)_ = 4.99, *P* < 0.005]; once stabilized, intake of chow and calories averaged ~35 g/kg and 110 kcal/kg, respectively.

Phase 2 was the 16-day period during which “cookie-experienced” rats developed hyperphagia for butter cookies. When exposed to the 1-h cookie-feeding sessions, “cookie-experienced” rats displayed indeed high intakes of butter cookies (Figure 3B) and calories from butter cookies (Figure 3D); both variables had stabilized by the third cookie-feeding session [cookie intake: *F*_(3, 69)_ = 28.79, *P* < 0.0001; calorie intake: *F*_(3, 69)_ = 27.58, *P* < 0.0001]; once stabilized, intake of butter cookies and intake of calories from butter cookies averaged ~25 g/kg and 130 kcal/kg, respectively.

ANOVA applied to data from Phase 2 indicated (i) a significant effect of time (16 consecutive days), but not of cookie exposure (“cookie-experienced” or “cookie-naive” rat groups), and a significant interaction between the 2 factors, on intake of chow [*F*_group(1, 58)_ = 2.88, *P* > 0.05; *F*_day(15, 870)_ = 5.89, *P* < 0.0001; *F*_interaction(15, 870)_ = 4.34, *P* < 0.0001] and (ii) significant effects of cookie exposure and time, as well as a significant interaction, on calories from chow [*F*_group(1, 58)_ = 11.06, *P* < 0.005; *F*_day(15, 870)_ = 5.95, *P* < 0.0001; *F*_interaction(15, 870)_ = 6.55, *P* < 0.0001]. Feeding behavior of cookie-experienced rats was characterized by periodic, marked reductions in intake of chow (Figure 3A) and calories from chow (Figure 3C) occurring every day after the cookie-feeding session; magnitude of these reductions averaged ~25% in comparison to intakes recorded in the same chow-feeding session in “cookie-naive” rats. Both variables returned to baseline levels by the second chow-feeding session following each cookie-feeding session. Conversely, intake of chow (Figure 3A) and calories (Figure 3C) in “cookie-naive” rats was stable over the entire 16-day period.

**Figure 4:**
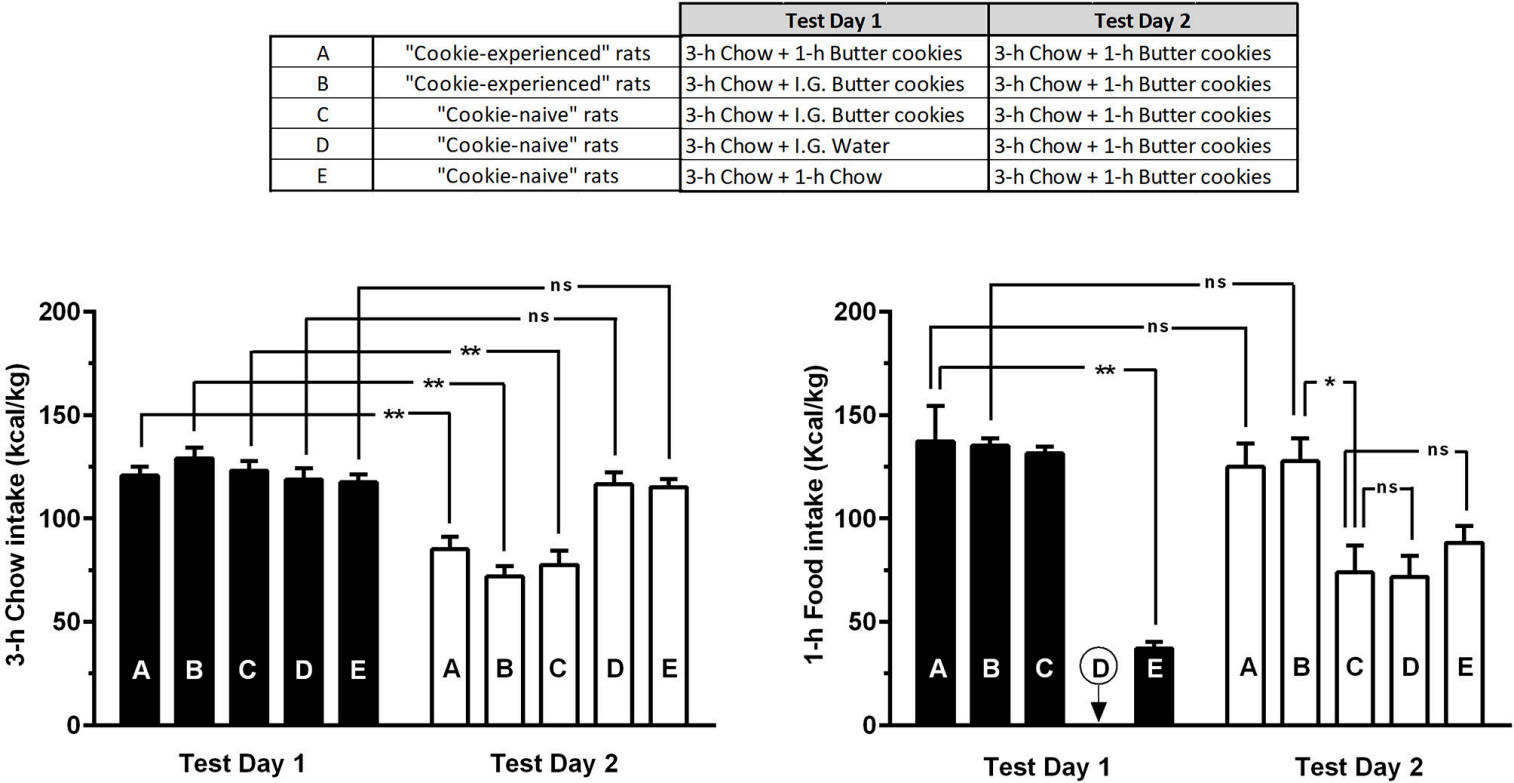
Intake of calories from chow or butter cookies in “cookie-experienced” and “cookie-naive” rats on the 2 test days of Phase 3 of Experiment 1. On Test Day 1, “cookie-experienced” rats were initially exposed to a 3-h chow-feeding session and then either exposed to a 1-h cookie-feeding session (Subgroup A) or given intragastrically an equivalent amount of cookies (Subgroup B); the day after (Test Day 2), both rat subgroups (A and B) were initially exposed to a 3-h chow-feeding session and then to a 1-h cookie-feeding session. On Test Day 1, “cookie-naive” rats were initially exposed to a 3-h chow-feeding session and then treated intragastrically with either cookies (Subgroup C) or water (Subgroup D) or exposed to a 1-h chow-feeding session (Subgroup E); the day after (Test Day 2), all 3 rat subgroups (C-E) were initially exposed to a 3-h chow-feeding session and then to a 1-h cookie-feeding session. Each bar is the mean ± SEM of *n* = 12 rats. *P < 0.005 and **P < 0.0001 (Šidák test). “ns” stands for “non-significant”.

ANOVA applied to data from Test Days 1 and 2 indicated significant effects of experimental condition (feeding regimen) and time (Test Days 1 and 2), as well as a significant interaction between the 2 factors, on intake of calories from chow in the 3-h feeding session [*F*_regimen(4, 55)_ = 4.98, *P* < 0.005; *F*_day(1, 55)_ = 87.84, *P* < 0.0001; *F*_interaction(4, 55)_ = 13.57, *P* < 0.0001] (Figure 4, left panel). *Post hoc* analysis indicated several relevant differences among rat subgroups. Specifically, all rat subgroups receiving butter cookies on Test Day 1 (Subgroups A-C in Figure 4) reduced—in a similar manner (30–45%)—their intake of calories from chow in the 3-h feeding session of Test Day 2 (Figure 4, left panel); this similarity in intake of calories from chow on Test Day 2 among rats having consumed cookies either freely or forcibly on Test Day 1 suggests that the repeatedly observed reduction in chow intake occurring the day after the 1-h cookie-feeding session was secondary to the day-before overload of cookie-derived calories and not to any self-restriction of chow feeding enacted in anticipation of cookie access. Additionally, intragastric administration of water (Subgroup D) or chow intake in an additional 1-h feeding session (Subgroup E) on Test Day 1 had no influence on the day-after intake of calories from chow (Figure 4, left panel).

ANOVA applied to data from Test Days 1 and 2 indicated a significant effect of experimental condition (feeding regimen), but not of time (Test Days 1 and 2), and a significant interaction between the 2 factors, on intake of calories from butter cookies (or chow) in the 1-h feeding session [*F*_regimen(4, 55)_ = 31.51, *P* < 0.0001; *F*_day(1, 55)_ = 3.01, *P* > 0.05; *F*_interaction(4, 55)_ = 19.63, *P* < 0.0001] (Figure 4, right panel). *Post hoc* analysis indicated several relevant differences among rat subgroups. Specifically, intake of calories from chow (deriving from 3.5 ± 0.7 g/kg chow; Subgroup E in Figure 4) was remarkable lower (~75%) than that of calories from cookies (deriving from 10.4 ± 1.1 g/kg butter cookies; Subgroup A) in the 1-h feeding session of Test Day 1 (Figure 4, right panel); this comparison further underlines the large intake of unnecessary calories in rats exposed to butter cookies. In both “cookie-experienced” rat subgroups (Subgroups A and B), intake of calories from cookies was virtually identical on Test Days 1 and 2 (Figure 4, right panel); this lack of difference suggests that rats overate cookies also on Test Day 2, in spite of the calorie overload of Test Day 1 (the same calorie overload that conversely inhibited chow intake). In all 3 “cookie-naive” rat subgroups (Subgroups C-E), intake of calories from cookies on Test Day 2 was highly similar and markedly lower than that recorded in both “cookie-experienced” rats (Subgroups A and B); these lower levels reflected the values commonly observed in the very first exposure of rats to a 1-h cookie-feeding session.

### Experiment 2: investigation of the effect of rimonabant

**Figure 5:**
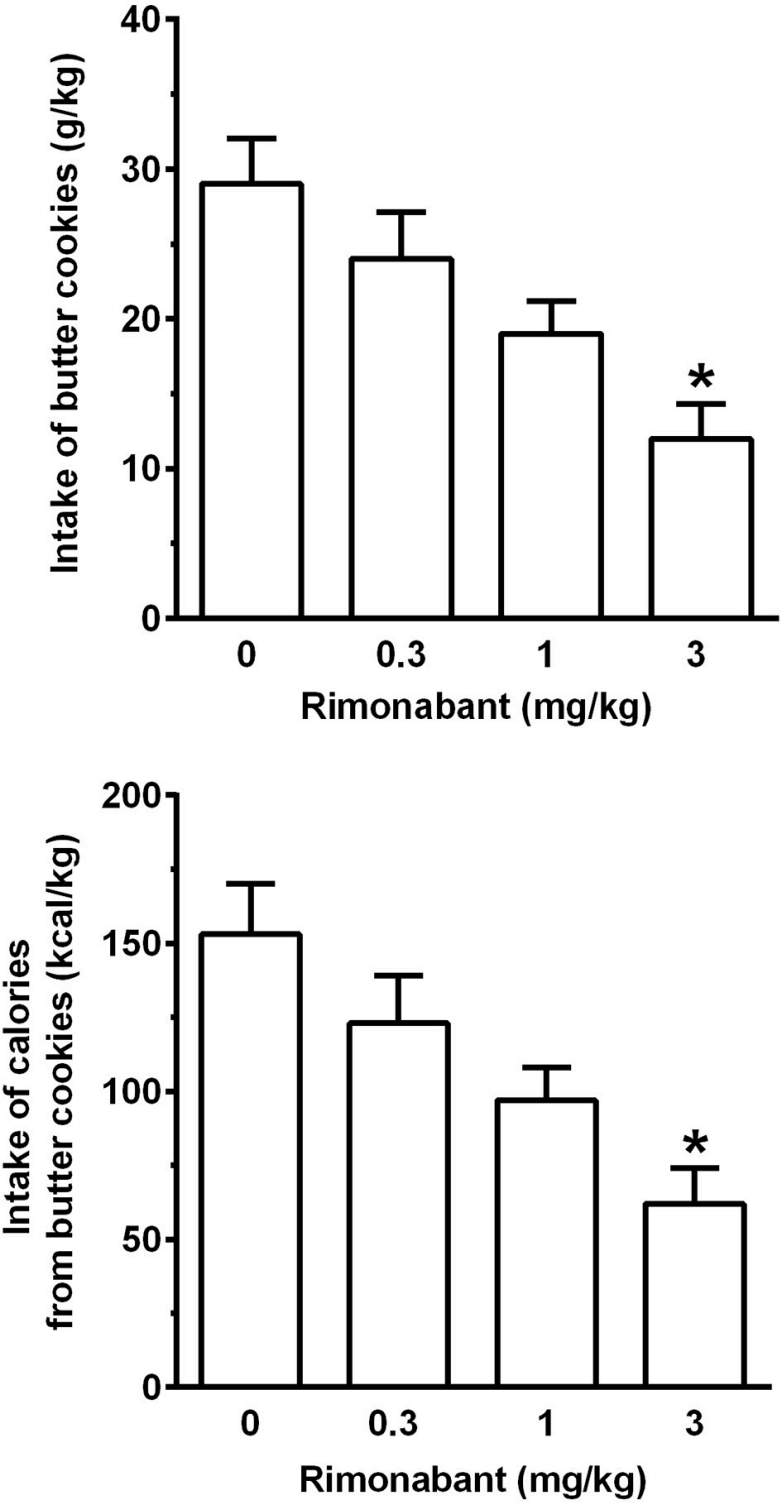
Effect of treatment with the cannabinoid CB_1_ receptor antagonist/inverse agonist, rimonabant, on intake of butter cookies **(top panel)** and calories from butter cookies **(bottom panel)** in rats habituated to a feeding regimen comprised of daily 3-h chow-feeding sessions and periodic 1-h cookie-feeding sessions (Experiment 2). Rimonabant was administered intraperitoneally immediately after the end of the 3-h chow-feeding session and 20 min before the start of the cookie-feeding session. All 4 doses of rimonabant were tested in each rat under a Latin-square design. Four consecutive daily 3-h chow-feeding sessions elapsed between test sessions with rimonabant. Each bar is the mean ± SEM of *n* = 12 rats. *P < 0.01 in comparison to the vehicle-treated rat group (Tukey test).

Treatment with rimonabant, administered immediately after chow removal and 20 min before cookie presentation, resulted in a dose-related reduction in intake of butter cookies [*F*_(3, 33)_ = 6.97, *P* < 0.005; Figure 5, top panel] and calories from butter cookies [*F*_(3, 33)_ = 7.16, *P* < 0.005] (Figure 5, bottom panel). In comparison to vehicle treatment, both variables were reduced by ~20, 35, and 60% after treatment with 0.3, 1, and 3 mg/kg rimonabant; statistical significance at the *post hoc* test was reached only by treatment with 3 mg/kg rimonabant.

Water intake during the 1-h cookie-feeding session averaged 11.8 ± 0.6, 8.4 ± 0.5, 8.5 ± 0.7, and 6.7 ± 0.7 ml/kg in the rat groups treated with 0, 0.3, 1, and 3 mg/kg rimonabant [*F*_(3, 33)_ = 5.71, *P* < 0.01], with a significant reduction, in comparison to vehicle treatment, in the rat group treated with 3 mg/kg rimonabant (*P* < 0.05, Tukey test).

### Experiment 3: investigation of the effect of SSA

**Figure 6:**
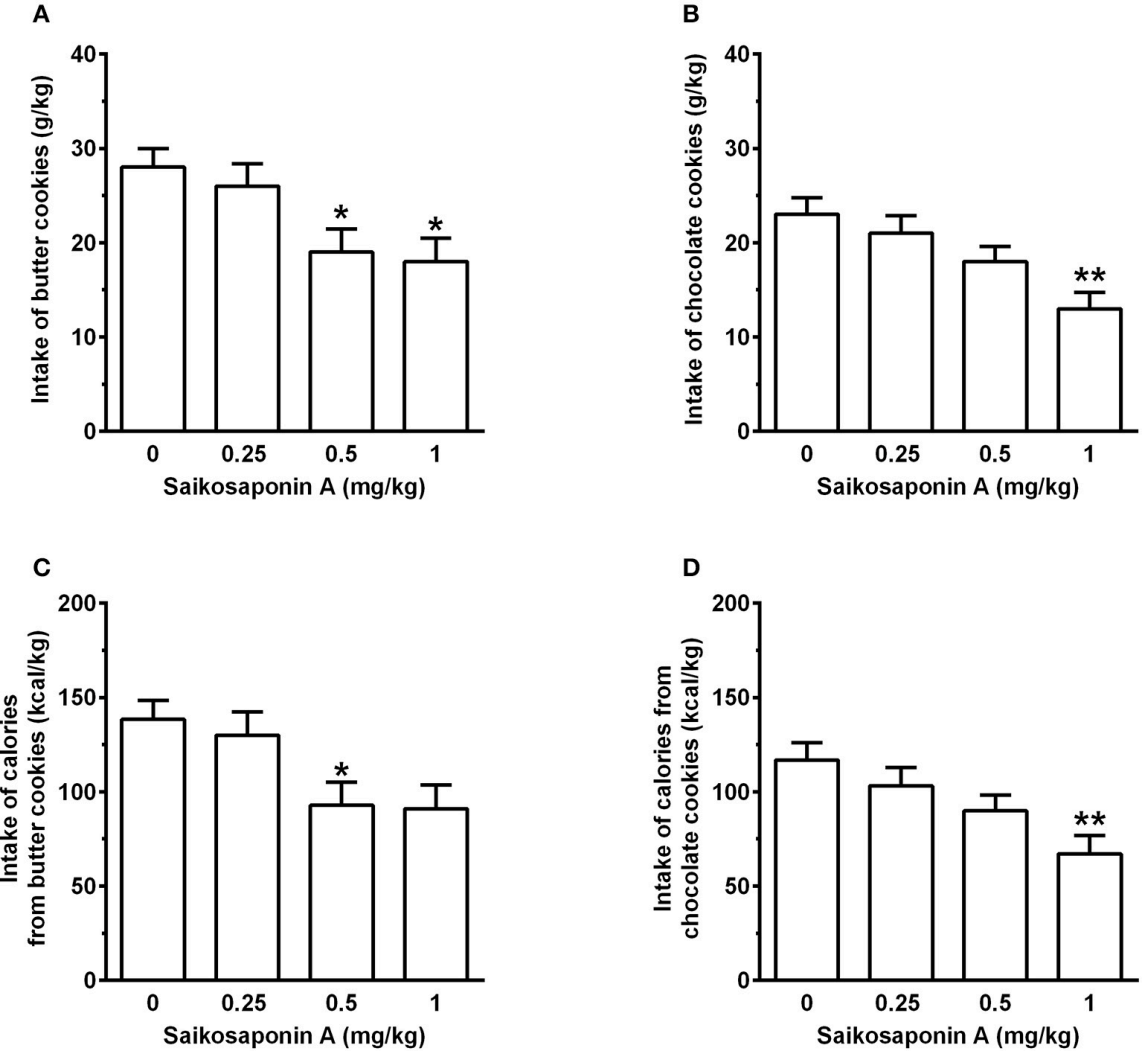
Effect of treatment with saikosaponin A (SSA) on intake of butter cookies **(A)** and chocolate cookies **(B)** as well as intake of calories from butter cookies **(C)** and chocolate cookies **(D)** in rats habituated to a feeding regimen comprised of daily 3-h chow-feeding sessions and periodic 1-h cookie-feeding sessions (Experiment 3). SSA was administered intraperitoneally immediately after the end of the 3-h chow-feeding session and 10 min before the start of the cookie-feeding session. All 4 doses of SSA were tested in each rat under a Latin-square design. Four consecutive daily 3-h chow-feeding sessions elapsed between test sessions with SSA. After the end of this first dose-curve assessment, rats previously exposed to butter cookies were exposed to chocolate cookies, and rats previously exposed to chocolate cookies were exposed to butter cookies. Rats of both groups underwent another test with SSA, again under a Latin-square design and with daily 3-h chow-feeding sessions elapsing between test sessions with SSA. Data were then cumulated to a final sample size of *n* = 24. Accordingly, each bar is the mean ± SEM of *n* = 24 rats. *P < 0.05 and **P < 0.01 in comparison to the vehicle-treated rat group (Tukey test).

Treatment with SSA, administered immediately after chow removal and 10 min before cookie presentation, resulted in a dose-related reduction in intake of butter cookies [*F*_(3, 69)_ = 4.01, *P* < 0.05] (Figure 6A) and calories from butter cookies [*F*_(3, 69)_ = 3.87, *P* < 0.05] (Figure 6C). In comparison to vehicle treatment, both variables were reduced by ~5, 35, and 35 after treatment with 0.25, 0.5, and 1 mg/kg SSA; statistical significance at the *post hoc* test was reached by treatment with 0.5 and 1 mg/kg SSA [with the only exception of a tendency toward a reduction after treatment with 1 mg/kg SSA on intake of calories from butter cookies (*P* = 0.053)]. Water intake during the 1-h butter cookie-feeding session averaged 10.4 ± 0.8, 10.4 ± 1.0, 10.2 ± 0.8, and 10.6 ± 0.8 ml/kg in the rat groups treated with 0, 0.25, 0.5, and 1 mg/kg SSA [*F*_(3, 69)_ = 0.09, *P* > 0.05].

Treatment with SSA also resulted in a dose-related reduction in intake of chocolate cookies [*F*_(3, 69)_ = 5.27, *P* < 0.005] (Figure 6B) and calories from chocolate cookies [*F*_(3, 69)_ = 5.62, *P* < 0.005] (Figure 6D). In comparison to vehicle treatment, both variables were reduced by ~10, 25, and 45% after treatment with 0.25, 0.5, and 1 mg/kg SSA; statistical significance at the *post hoc* test was reached only by treatment with 1 mg/kg SSA. Water intake during the 1-h butter cookie-feeding session averaged 10.7 ± 0.6, 9.4 ± 0.7, 9.8 ± 0.7, and 9.4 ± 0.7 ml/kg in the rat groups treated with 0, 0.25, 0.5, and 1 mg/kg SSA [*F*_(3, 69)_ = 0.97, *P* > 0.05].

## Discussion

The present study employed an experimental procedure intended to model overeating of and loss of control over a highly tasty, sweet food after a large meal. To this end, rats were exposed every day to 21 h of food deprivation and 3 h of free access to regular chow; rats became rapidly habituated to feed sufficient chow over the daily brief feeding sessions to even increase their body weight (Figure 2). Every 4 days, rats were exposed to 1-h sessions with access to highly palatable butter or chocolate cookies. These sessions occurred immediately after the end of the 3-h chow-feeding period, with the intent of maximizing overeating of unnecessary calories. Each time cookies were made available, and despite being fed, rats displayed high consumptions of cookies. Vernacularly speaking, we might define this experimental procedure as a rat model of overindulgence for sweet food.

Calories obtained from cookies (~140 kcal/kg) in one h were even higher than those obtained from chow (~120 kcal/kg) in 3 h, suggestive of hyperphagic behavior and intake of remarkable amounts of unnecessary calories. The magnitude of such overload of unnecessary calories is further evidenced by the ~4:1 ratio on calories from chow consumed by the rat subgroup exposed to an additional 1-h feeding session with chow instead of butter cookies (Figure 4, right panel). Calorie overload from cookies was sufficiently large to invariably result in marked reductions in chow intake in the following daily feeding session, regardless of the 20 h of food deprivation elapsing between cookie removal and chow presentation. The impact of overload of cookie-derived calories on day-after chow intake is well depicted by the sawtooth-like pattern of chow intake illustrated in Figure 3A: chow intake decreased by ~25% in each daily chow-feeding session occurring the day after a cookie-feeding session.

The effect of overload of cookie-derived calories on day-after chow intake is also confirmed by the results of the experiment in which an amount of butter cookies equivalent to that usually consumed freely by rats was administered forcibly and intragastrically: the latter regimen resulted indeed in an identical reduction in chow intake in the day-after chow-feeding session (Figure 4, left panel). These results also tend to exclude that the day-after reduction in chow intake could be secondary to a self-restriction of chow feeding enacted in anticipation of cookie access, i.e. a mechanism initially hypothesized on the basis of results of a recent study demonstrating an anticipatory self-hypophagia of regular chow in rats kept under a regimen of mild food deprivation and then exposed to 2 brief, contiguous periods of access to chow first and chocolate pellets later (11). In the present study, chow intake on Test Day 2 was reduced in cookie-naive rats given cookies intragastrically (Figure 4, left panel) to an extent virtually identical to that observed in cookie-experienced rats. This additional comparison provides further support to the hypothesis that the day-after reduction in chow intake was exclusively due to the overload of cookie-derived calories and was not influenced, even minimally, by any environmental cue potentially predictive of cookie availability, that could have contributed to an anticipatory self-hypophagia.

Conversely, the overload of cookie-derived calories did not affect day-after cookie intake, as rats consumed identical amounts of butter cookies in two consecutive daily cookie-feeding sessions (Figure 4, right panel). In other words, cookie intake in the second test session was totally unaffected by the day-before calorie overload (the same calorie overload that conversely had produced a marked impact on chow intake). The resistance, or inflexibility, of cookie intake to the previous calorie overload may be interpreted as a sign of behavioral dependence of rats upon palatable food.

The cookie overconsumption generated by this protocol was pharmacologically manipulable, as suggested by the results of the experiment testing the cannabinoid CB_1_ receptor antagonist/inverse agonist, rimonabant. Rimonabant has repeatedly been reported to suppress several behaviors motivated by palatable food in rats [e.g., (12–16)], making it an appropriate reference compound for use in validating the experimental procedure used in the present study, as well as providing a comparison to the tested compound (SSA). In the present study, treatment with rimonabant—administered after the chow-feeding session and before presentation of butter cookies—resulted in a marked and dose-related reduction in cookie intake (Figure 5). These data closely replicate those previously collected in a similar experimental paradigm (19) and confirm the ability of rimonabant to reduce food intake in rodent models of overeating.

The herbal ingredient SSA, administered at the end of the chow-feeding session and ~10 min before access to cookies, reduced the intake of both butter and chocolate cookies (Figure 6). At the highest doses tested (1 mg/kg), reduction in intake of butter and chocolate cookies—compared to vehicle treatment—averaged ~35 and 45%, respectively.

The dose range of SSA tested in the present study is known not to induce any sedative or motor-incoordinating effects in Wistar rats (7). It can therefore be excluded that the reducing effect of SSA on butter and chocolate cookies was secondary to unspecific effects that would have hampered the rat capability to grasp and consume the cookies.

The capability of SSA to reduce cookie overeating at doses as low as 0.5 and 1 mg/kg is suggestive of SSA potency in *in vivo* assays. The notion of SSA potency is further strengthened when considering that SSA—because of its chemical structure constituted by an aglycone and a saccharide chain—is a heavy molecular weight compound (its molecular weight is indeed equal to 780.98).

The results of the present study are in line with those of a recent study demonstrating that treatment with SSA potently and effectively suppressed operant self-administration of and reinstatement of seeking behavior for a highly palatable chocolate-flavored beverage in rats (7). SSA appears therefore to be able to decrease the reinforcing and motivational properties (7), seeking behavior after withdrawal (7), and hyperphagia (present study) of palatable food in rats.

Studies are now needed to identify the mechanisms underlying SSA actions on the above behaviors. The suppressing effects of SSA on morphine (2), cocaine (3), and alcohol (4) self-administration in rats was blocked, at least partially, by pretreatment with the GABA_B_ receptor antagonist, SCH50911, suggesting the involvement of the GABA_B_ receptor in the neural substrate mediating the *anti*-addictive properties of SSA. The contribution of the GABA_B_ receptor to the mechanisms regulating food intake is matter of considerable debate, with the majority of studies reporting an increase, rather than a decrease, in food intake in rodents after treatment with GABA_B_ receptor agonists [e.g., (20–23)]; thus, although not experimentally addressed, it seems to be unlikely that the suppressing effects of SSA on behaviors motivated by palatable food are mediated by the GABA_B_ receptor. A recent study (24), reporting the reducing effect of acutely administered SSA on chow intake in rats, hypothesized an underlying 5-HT_2C_-mediated mechanism: this hypothesis was based on (a) the agonistic activity of SSA on 5-HT_2C_ receptor described in that study (24) and (b) the known anorectic properties of 5-HT_2C_ receptor agonists in rodents [see (25, 26)]. Whether the 5-HT_2C_ receptor is part of the neural substrate mediating the reducing effect of SSA on palatable food is an intriguing hypothesis that merits to be assessed experimentally.

As all studies conducted to date have employed male rats, additional experiments are needed to investigate whether, and to what extent, the suppressing effects of SSA on behaviors motivated by palatable food extend to female rats. This research question is of relevance also in light of the recent recommendation of considering sex as a biological variable in preclinical studies (27, 28).

In conclusion, the results of the present study indicate that treatment with the plant derivative, SSA, potently and effectively reduced overeating of highly palatable butter and chocolate cookies in pre-fed rats exposed to an experimental procedure thought to model human intake of large amounts of unnecessary calories. These data complement with those of recent studies indicating the ability of SSA to reduce regular food intake (24) as well as the reinforcing and motivational properties of a chocolate-based highly palatable beverage (7) in rats and, at a wider range, those of studies reporting the suppressing effect of SSA on the reinforcing and motivational properties of different drugs of abuse in rats (2–4). Further studies are needed to extend the anorectic profile of SSA to other food-related behaviors and to possibly identify the mechanisms underlying the anorectic effects of SSA.

## Author contributions

GC, GG, and PM conceived the study and designed the experiment. GC, MC, and PM set up the experimental procedure. Y-WC, JL, and HK prepared and analyzed the plant extract. GC, FF, and PM conducted the experiment. FF and PM analyzed the data. GC drafted the manuscript. All authors contributed to and approved the final draft of the manuscript.

### Conflict of interest statement

The authors declare that the research was conducted in the absence of any commercial or financial relationships that could be construed as a potential conflict of interest.
